# The miR165/166 Mediated Regulatory Module Plays Critical Roles in ABA Homeostasis and Response in *Arabidopsis thaliana*

**DOI:** 10.1371/journal.pgen.1006416

**Published:** 2016-11-03

**Authors:** Jun Yan, Chunzhao Zhao, Jianping Zhou, Yu Yang, Pengcheng Wang, Xiaohong Zhu, Guiliang Tang, Ray A. Bressan, Jian-Kang Zhu

**Affiliations:** 1 Shanghai Center for Plant Stress Biology, and Center for Excellence in Molecular Plant Sciences, Chinese Academy of Sciences, Shanghai, China; 2 Department of Horticulture and Landscape Architecture, Purdue University, West Lafayette, Indiana, United States of America; 3 Department of Biological Sciences, Michigan Technological University, Houghton, Michigan, United States of America; National University of Singapore and Temasek Life Sciences Laboratory, SINGAPORE

## Abstract

The function of miR165/166 in plant growth and development has been extensively studied, however, its roles in abiotic stress responses remain largely unknown. Here, we report that reduction in the expression of miR165/166 conferred a drought and cold resistance phenotype and hypersensitivity to ABA during seed germination and post-germination seedling development. We further show that the ABA hypersensitive phenotype is associated with a changed transcript abundance of ABA-responsive genes and a higher expression level of *ABI4*, which can be directly regulated by a miR165/166 target. Additionally, we found that reduction in miR165/166 expression leads to elevated ABA levels, which occurs at least partially through the increased expression of *BG1*, a gene that is directly regulated by a miR165/166 target. Taken together, our results uncover a novel role for miR165/166 in the regulation of ABA and abiotic stress responses and control of ABA homeostasis.

## Author Summary

Functions of miRNAs in plant development and stress responses have been extensively studied. However, little is known about how a miRNA may perform critical functions in both plant development and abiotic stress responses. One well-known miRNA, miR165/166, has critical roles in plant development. In this study, we show that this miRNA also has important functions in ABA and abiotic stress responses. Since the expression level of miR165/166 can be reduced to different extents using short tandem target mimicry (STTM), in the present work, we used STTM165/166 transformants with moderate developmental phenotype to examine its potential role in abiotic stress responses. Our results show that miR165/166 plays critical roles in drought and cold stress resistance as well as in ABA responses. Our work reveals that miR165/166-mediated regulatory module is linked with ABA responses and homeostasis through ABI4 and BG1.

## Introduction

The phytohormone abscisic acid (ABA) plays critical roles in plant growth and development, such as seed maturation, seed germination, seedling growth, stomatal movement, as well as plant responses to abiotic and biotic stress, including drought, salinity, cold and pathogen infection [1–4]. The fluctuation of cellular ABA levels, which are determined by biosynthetic and catabolic pathways, allow plants to cope with physiological and environmental conditions [5–7]. De novo ABA biosynthesis from carotenoids is the primary pathway to produce ABA [7]. Many genes involved in this pathway have been identified, such as *ABA1*, *ABA2*, *ABA3*, *NCED3* and *AAO3* [8, 9]. An additional biosynthetic pathway occurs through hydrolysis of Glc-conjugated ABA (abscisic acid-glucose ester [ABA-GE]) to ABA by two glucosidases, AtBG1 and AtBG2, which localize to the ER and vacuole, respectively [10, 11]. Hydroxylation and conjugation are catabolic pathways that mediate the fine-tuning of ABA levels. Members of the cytochrome P450 family, CYP707A1 to CYP707A4, control the hydroxylation reaction, and ABA uridine diphosphate glucosyltransferase (UGT) catalyzes the conjugation of ABA with Glc to produce ABA-GE [12–17].

Plant responses to ABA is mediated by a network of signaling pathways. In the core pathway, ABA is perceived by the ABA receptors, PYRABACTIN RESISTANCE1 (PYR1)/PYR1-likes/REGULATORY COMPONENT OF ABA RECEPTORs (RCARs) [18, 19]. Once bound to ABA, PYLs will recruit PROTEIN PHOSPHOSTASE 2C (PP2C) [20] and form a PYR/RCAR-PP2C complex to inhibit the PP2C activity, thereby activating the SNF1-RELATED PROTEIN KINASE2 (SnRK2) kinases [21–23]. The activated SnRK2s phosphorylate downstream effector proteins including the AREB/ABF-type basic/region leucine zipper (bZIP) transcription factors, which control the expression of many ABA-responsive genes [24, 25]. Among these transcription factors, ABA INSENSITIVE 3 (ABI3), ABI4 and ABI5 are essential regulators in the control of seed germination and early seedling growth [26–32].

A class of single-stranded RNAs that are 20–22 nucleotides in length and are referred to as microRNA (miRNAs) can regulate gene expression at post-transcriptional levels through specific base-pairing to target messenger RNAs [33]. miRNAs play critical roles in plant development, such as phase transition, pattern formation and morphogenesis [34]. miRNAs also play crucial roles in biotic and abiotic stress responses [35–38].

Additionally, more and more evidence is revealing that miRNAs are involved in hormonal responses. miR159 targets several *MYB* transcription factors, such as MYB33, MYB65 and MYB101, which interact with GA-response elements and control anther development and flowering time under short days [39, 40]. Disruption of the miR159-mediated repression of *MYB33* and *MYB101* alters responses to ABA during seed germination [41]. The auxin response pathway is also regulated by miRNAs. Proper regulation of *Auxin Response Factor 10* (*ARF10*), *ARF16* and *ARF17* by miR160 is required for both shoot and root development [42–44]. *ARF6* and *ARF8* are targeted and negatively regulated by miR167 [45, 46]. Expression of a miR167-resistant *ARF6* or *ARF8* gene results in ovule and anther development defects [47]. miR167 could also target *IAA-Ala Resistant3* (*IAR3*), which converts an inactive form of auxin to bioactive auxin [48]. miR390 guides the generation of trans-acting siRNAs, which target *ARF2*, *ARF3* and *ARF4* that are required for the proper establishment of adaxial-abaxial identity of lateral organs and vegetative phase transition [49–52]. The *NAC1* transcription factor is targeted by miR164 and acts on lateral root development through regulating auxin responses [45, 53–56]. miR393 targets auxin receptor *TIR1* and closely related F-box genes [46, 57]. In addition, miR319-mediated regulation of *TCP4* is required for the biogenesis of jasmonic acid through the modulation of *LIPOXYGENASE2* (*LOX2*) [58].

miR165/166 is one of the most extensively studied miRNAs, which have been shown to be involved in plant development. miR165/166 targets the Class III homeodomain leucine zipper family of transcription factor genes, including *PHBULOSA* (*PHB*), *PHVOLUTA* (*PHV*), *REVOLUTA* (*REV*), *ATHB-8* and *ATHB-15*, which are required for the promotion of adaxial identity of lateral organs [59–62]. Recent work revealed that *REV* could directly regulate the expression of auxin biosynthetic enzymes *TAA1* and *YUCCA5* (*YUC5*), which in turn influence free auxin levels, and this was shown to be required for the shade-avoidance response [63]. The cytokinin (CK) biosynthesis gene *ISOPENTENYL TRANSFERASE 7* (*IPT7*) was found to be the direct target of PHB, and the direct activation of *IPT7* by PHB was shown to control the root meristem differentiation regulatory network [64].

Here we present evidence for an important role for miR165/166 in the regulation of ABA and abiotic stress responses and the maintenance of ABA homeostasis. We show that disruption of miR165/166-mediated repression of its targets through reducing miR165/166 expression levels leads to a drought and cold resistance phenotype and ABA hypersensitivity during and after seed germination. We found that ABI4 acts downstream of a miR165/166-mediated pathway and could be directly regulated by a miR165/166 target. We also discovered that miR165/166-mediated negative regulation of its targets is essential for maintaining ABA homeostasis at least partly through modulating the expression of *BG1*, which converts inactive ABA to active ABA. Our study links the miR165/166-mediated regulatory module to the ABA regulatory network and demonstrates a critical role for the miRNA in ABA responses and homeostasis.

## Results

### STTM165/166 displays a drought and cold resistance phenotype

To determine whether the miR165/166 mediated network plays important roles in response to abiotic stress, the previously reported stable transgenic *Arabidopsis* STTM165/166-31nt plants [65], in which the expression of miR165/166 is dramatically reduced, were used in stress resistance tests.

**Fig 1:**
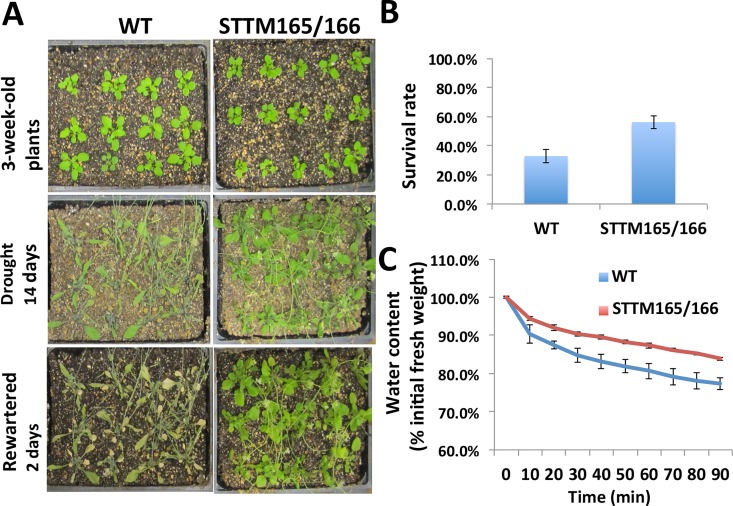
STTM165/166 plants exhibit drought stress resistance phenotype. (A) Drought resistance phenotype. 3-week-old plants (upper panel) were grown under the same conditions but without irrigation for 14 days (middle panel), and then rewatered for 2 days (lower panel). (B) Quantification of the survival rate. Forty plants of wild type and STTM165/166 were used in each experiment, and the survival rate was calculated from the results of four independent experiments. (C) Water loss assay. Aerial parts of 3-week-old plants were detached and weighed at the indicated time points. Water content at any time point was calculated as percentage of the fresh weight at time zero. Data were derived from four independent experiments (±SD).

We compared the phenotype of wild type and STTM165/166 plants under drought conditions. When water was withheld from 3-week-old plants for up to 2 weeks, wild type plants severely wilted and displayed injury and reduced growth. In contrast, STTM165/166 plants appeared much healthier and less affected by the limited water (**Fig 1A**). When the wilted wild type and STTM165/166 plants were re-watered, only a small proportion of the wild type plants survived and continued to grow. However, a substantial proportion of STTM165/166 plants recovered (**Fig 1A and 1B**). Altered sensitivity to drought stress in plants is often caused by an altered rate of water loss from leaves. Consequently, we analyzed the water loss rate and found that detached wild type leaves lost water at a faster rate than STTM165/166 leaves (**Fig 1C)**.

**Fig 2:**
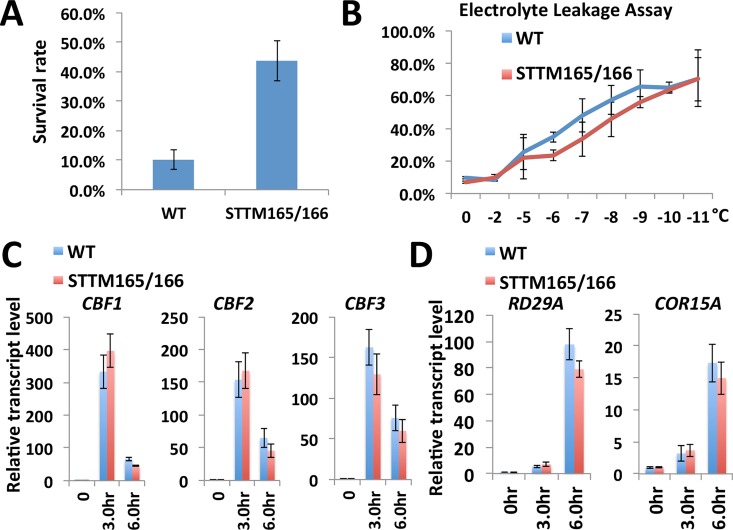
STTM165/166 plants display cold tolerance phenotype. (A) Comparison of the survival rates of freezing-treated wild type and STTM165/166 seedlings after 5 days of recovery at 23°C. (B) Leakage of electrolytes from excised leaves of wild type and STTM165/166 plants exposed to indicated freezing temperatures. Wild type and STTM165/166 plants were grown under normal conditions for three weeks and then subjected to cold acclimation (4°C for 7 days) before freezing treatment. (C) Comparison of the expression of *CBF* genes in wild type and STTM165/166 under cold treatment. (D) Comparison of the expression of cold responsive genes in wild type and STTM165/166 under cold treatment.

Interestingly, STTM165/166 is also more resistant to freezing temperatures compared with wild type based on freezing survival assay and cold-induced electrolyte leakage assay **(Fig 2A and 2B)**. Given that *CBF* genes play critical roles in freezing tolerance, we analyzed the expression of these genes to test whether miR165/166 mediated regulation of freezing tolerance occurs through modulating CBF factors. However, no substantial difference in the expression levels and patterns of *CBF1-3* under cold treatment was detected between wild type and STTM165/166 plants (**Fig 2C**). The expression of known *CBF* downstream genes, such as *RD29A* and *COR15A*, was also analyzed (**Fig 2D**) and we found that the transcript levels of these genes in STTM165/166 in response to cold stress are similar to that of wild type. These results indicate that miR165/166 may modulate freezing tolerance through CBF-independent factors.

### Deregulation of miR165/166 results in an ABA response phenotype

**Fig 3:**
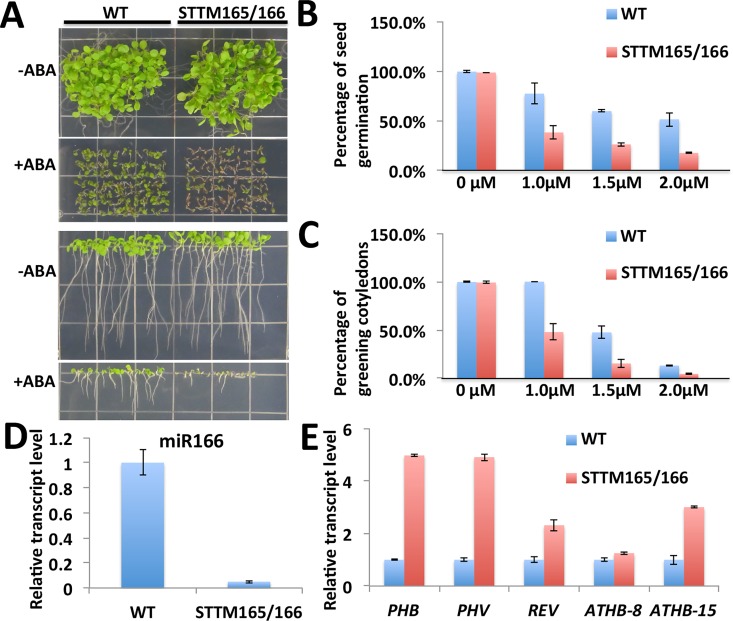
STTM165/166 plants are hypersensitive to ABA during seed germination and early seedling development. (A) The phenotype of wild type and STTM165/166 plants grown on MS medium supplemented with 1.0 μM ABA. Seedlings were photographed 9 days after stratification. (B, C) Germination and cotyledon greening analysis of the STTM165/166 and wild type plants in response to different concentrations of ABA (0, 1.0, 1.5 and 2.0 μM). Germination was scored at 4 days and greening was scored at 9 days after stratification. Three independent experiments were performed, and >100 seeds for each treatment were used for each experiment. Values are means ± standard deviation. (D, E) Quantitative RT-PCR analysis of the expression of both mature miR165/166 and its targets at seedling stage. Three independent experiments were performed, and values are means ± standard deviation.

We also tested the response of STTM165/166 to ABA. Without ABA treatment, there was no significant difference in the seed germination and cotyledon greening between wild type and STTM165/166 (**Fig 3A**). However, when the wild type and STTM165/166 seeds were sown on MS medium supplemented with ABA, we found that STTM165/166 was hypersensitive to ABA during seed germination. A delay of cotyledon greening was also observed for STTM165/166 plants (**Fig 3A, 3B and 3C** and **S1 Fig**). We examined the expression of miR165/166 and its targets in STTM165/166 at this early developmental stage with or without ABA treatment by qRT-PCR analysis, and found that the levels of mature miR165/166 were indeed dramatically reduced (**Fig 3D** and **S2 Fig**), and all the five target RNAs examined were elevated to different extents (**Fig 3E** and **S2 Fig**). These results indicate that blocking the full function of miR165/166 disturbs ABA responses. We also tested the ABA response of mutants of miR165/166 target genes, but did not observe a significant difference with that of the wild type (**S3 Fig**).

### The miR165/166 mediated regulatory module affects the expression of ABA- responsive genes

**Fig 4:**
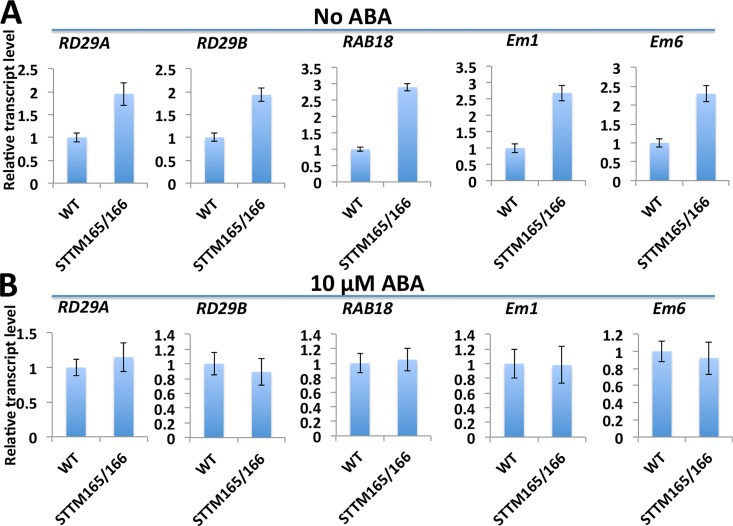
Quantitative RT-PCR analysis of ABA-responsive genes in wild type and STTM165/166 seedlings. (A) The expression of ABA-responsive genes was analyzed in wild type and STTM165/166 2-day-old seedlings grown on MS medium. Three independent experiments were performed and values are means ± standard deviation. (B) The expression of ABA-responsive genes was analyzed in wild type and STTM165/166 2-day-old seedlings grown on MS medium with 10 μM ABA. Three independent experiments were performed and values are means ± standard deviation.

The ABA-related phenotype that results from the compromised miR165/166 function indicates that a miR165/166 mediated regulatory module may affect ABA responses. To establish the molecular link between a miR165/166 mediated regulatory module and an ABA mediated regulatory network, we first compared expression of ABA-responsive genes, such as *RESPONSIVE TO DESSICATION 29A* (*RD29A*), *RD29B*, *RAB18*, *EM1* and *EM6* in wild type and STTM165/166 plants. Interestingly, without ABA treatment, the transcript levels of these genes were upregulated to different extents in STTM165/166 plants (**Fig 4A**). However, the difference in the expression of these genes disappeared when exogenous ABA was applied (**Fig 4B**). When seedlings were treated with 50 μM ABA for different time periods, there were still no significant differences in the transcript levels of ABA-responsive genes between wild type and STTM165/166 plants (**S4 Fig**).

### Targets of miR165/166 directly regulate the expression of *ABI4*

**Fig 5:**
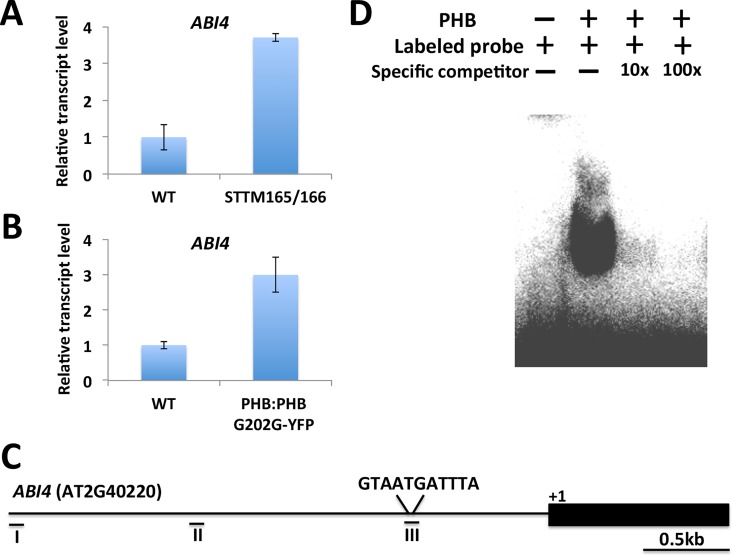
PHB promotes *ABI4* expression by directly binding to its promoter. (A) *ABI4* expression was analyzed in wild type and STTM165/166 2-day-old seedlings using qRT-PCR. (B) *ABI4* expression was analyzed in *PHB*:*PHB G202G-YFP* lines using qRT-PCR. (C) Analysis of *ABI4* promoter. A 3.0kb fragment upstream of ATG was chosen for the promoter analysis. (D) EMSA assay showed that PHB binds to an *ABI4* promoter region. Labeled probe of the *ABI4* promoter region was incubated with GST-PHB fusion protein. For the competition test, a non-labeled probe was added at 10-fold and 100-fold concentrations.

Since the expression of ABA-responsive genes was upregulated in STTM165/166 under normal conditions, we speculated that ABA signaling may be activated or endogenous ABA level is altered. We examined the expression of core components of ABA signaling pathway, such as PYLs (PYR1, PYL1, PYL2, PYL4, PYL5), SnRK2s (SnRK2.2, SnRK2.3, SnRK2.6), ABI1, ABI2 and HAB1. No significant difference in the expression of these genes was found between wild type and STTM165/166 **(S5 Fig)**. We also compared the expression of these genes in wild type and STTM165/166 seedlings treated with 50 μM ABA for different time periods, and no significant difference was detected **(S6, S7** and **S8 Figs)**. Since *ABI3*, *ABI4* and *ABI5* are central regulators in the control of ABA- responsive genes, we determined the effect of knockdown miR165/166 on the expression of these genes. We found that the expression of *ABI4* was substantially increased in STTM165/166 under normal conditions (**Fig 5A**). We then tested the expression of *ABI4* in *PHB*:*PHB G202G-YFP* lines expressing a miRNA-resistant version of *PHB* fused to GFP driven by the *PHB* promoter. Interestingly, we found that *ABI4* transcripts accumulated to higher levels in the tagged lines compared with that of wild type (**Fig 5B**). Bioinformatic analysis revealed that a typical HD-ZIPIII binding consensus sequence exists in the *ABI4* promoter region (**Fig 5C**), and this prompted us to determine whether *ABI4* could be directly regulated by a miR165/166 targeted HD-ZIPIII. Thus, we conducted an EMSA assay, and found that PHB protein could bind to the region containing the typical HD-ZIPIII binding consensus sequence (**Fig 5D**). This indicates that a miR165/166 target can directly modulate *ABI4* expression.

### ABA homeostasis is altered in STTM165/166

**Fig 6:**
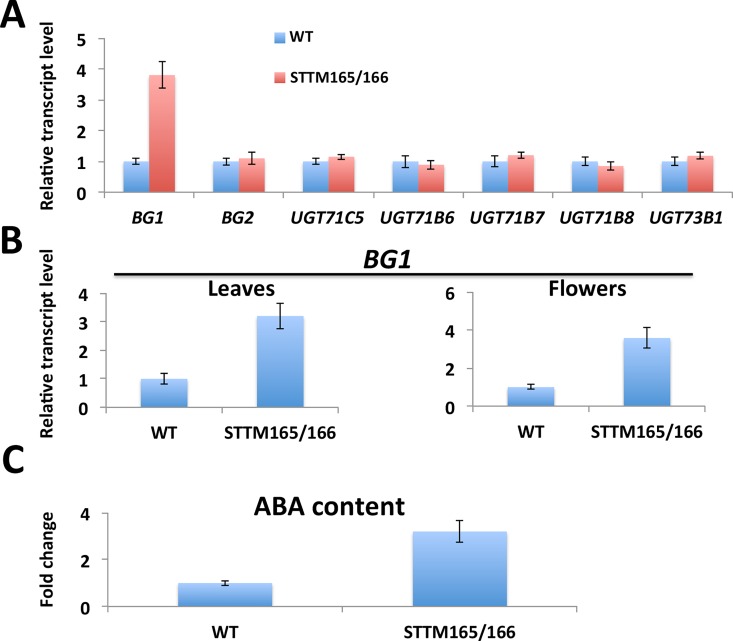
ABA content is altered in STTM165/166 plants. (A) Quantitative RT-PCR analysis of the expression of genes involved in ABA conjugation and de-conjugation. (B) Quantitative RT-PCR analysis of *BG1* expression in various tissues of wild type and STTM165/166 plants. (C) Comparison of the ABA content between wild type and STTM165/166 plants.

We also examined the expression of genes involved in the ABA homeostasis pathway. We could not detect any significant difference in the transcript level of any gene involved in de novo ABA biosynthesis, such as *ABA1* and *NCED3*, between wild type and STTM165/166 **(S9 Fig)**. In addition to genes involved in ABA de novo synthesis, genes required for ABA conjugation or deconjugation also affect ABA homeostasis. We then checked the expression of genes involved in this pathway. Interestingly, we found that the expression of *BG1* was dramatically elevated in STTM165/166 seedlings compared with that of wild type (**Fig 6A**), but the expression of *UGT* genes was not altered (**Fig 6A**). We also found that the upregulation of *BG1* in STTM165/166 was not limited to the seedling stage. The transcript level of *BG1* was also higher in STTM165/166 leaves and flowers compared with that of wild type (**Fig 6B**).

To examine the effect of the altered expression of these genes on ABA levels in STTM165/166, we measured the content of ABA by ELISA using an anti-ABA antibody [10]. We found that the ABA content in STTM165/166 plants was approximately 3 fold of that in wild type (**Fig 6C**). This indicates that regulation of the *BG1* gene mediated by the miR165/166 regulatory module contributes to changes in ABA content.

To determine whether the upregulated *BG1* expression might contribute to the drought resistance and reduced water loss phenotypes of STTM165/166 plants, we generated STTM165/166 plants in *bg1-2* mutant background by crossing STTM165/166 plants and *bg1-2* mutant plants, and we found that the drought resistance and reduced water loss phenotypes of STTM165/166 plants were partially suppressed by *bg1-2* (**S10 Fig**). These indicate that the upregulation of *BG1* in STTM165/166 accounts at least partially for its abiotic stress phenotypes.

### miR165/166 targets directly regulate the expression of *BG1*

**Fig 7:**
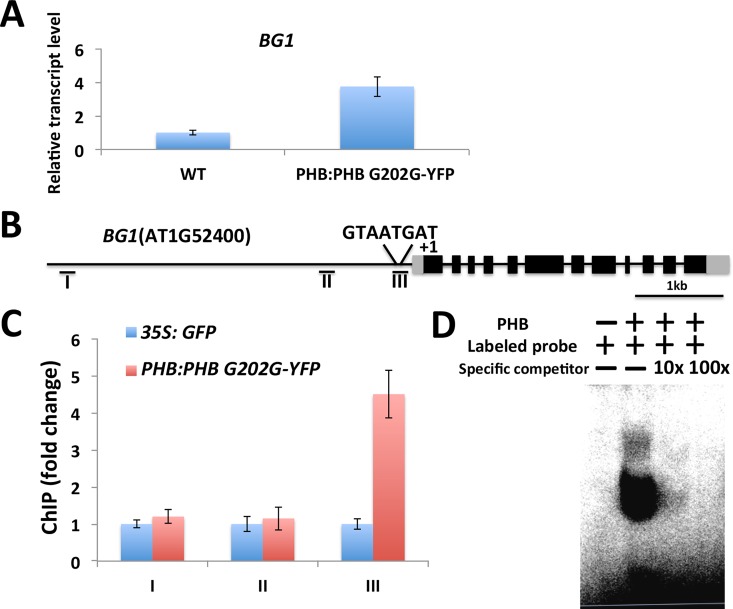
PHB directly upregulates the expression of *BG1*. (A) *BG1* expression was analyzed using qRT-PCR in *PHB*:*PHB G202G-YFP* lines. (B) Analysis of *BG1* promoter. The diagram shows a 4.0kb fragment upstream of the first ATG codon. (C) ChIP-qPCR was performed using specific primers corresponding to different promoter regions. (D) EMSA assay showed that PHB binds to the *BG1* promoter region. Labeled probes of the *BG1* promoter region were incubated with GST-PHB fusion protein. For the competition test, a non-labeled probe was added at 10-fold and 100-fold concentrations.

Since the expression of *BG1* was enhanced in STTM165/166, we next investigated its expression in *PHB*:*PHB G202G-YFP* lines to determine whether higher expression of *PHB* could also affect the expression of *BG1*. We found that the expression of *BG1* was upregulated in the *PHB*:*PHB G202G-YFP* line (**Fig 7A**). To determine whether PHB is directly associated with the *BG1* promoter, we first analyzed the sequence of the *BG1* promoter and found that it contains a PHB recognition motif (**Fig 7B**). A ChIP assay was then performed using *PHB*:*PHB G202G-YFP* lines and this showed that one region of the promoter was highly enriched relative to the *35S*:*GFP* control (**Fig 7C**). The enriched region contains the PHB recognition motif. Additionally, EMSA assay further confirmed that PHB protein could bind to the enriched region (**Fig 7D**). These findings indicate that *BG1* is also a direct target of PHB.

## Discussion

Unlike some miRNAs, such as miR160, miR167 and miR393, which directly target and regulate the expression of key components of the auxin response pathway, the miR165/166 targets themselves are not major components of hormone response pathways but they regulate the transcription of important components of hormone pathways. Recent work showed that REV could directly modulate auxin biosynthetic gene expression and is involved in the shade-avoidance response pathway [63], whereas PHB directly activates the CK biosynthesis gene *IPT7* and is integrated into the root meristem differentiation regulatory network [64]. Here we provide evidence that the miR165/166-*PHB* module is involved in regulating ABA homeostasis. The expression of *BG1* could be directly promoted by PHB. Therefore, upregulation of the miR165/166 target gene expression caused by compromised miR165/166 function results in the increased expression of *BG1*, which in turn further modulates ABA homeostasis.

Proper regulation of miR165/166 is important for normal ABA responses. Once miR165/166 is repressed, its repression on target genes will be released, and the upregulated expression of miR165/166 targets will directly promote the accumulation of *ABI4*, which in turn activates downstream ABA responsive genes. Meanwhile, the increased miR165/166 targets could also upregulate the expression of the *BG1* gene, which at least in part contributes to the elevation of ABA content in STTM165/166. Thus, the ABA hypersensitivity phenotype of STTM165/166 during seed germination and post-germination stages might be attributed to both the higher levels of active ABA and stronger ABA response caused by higher expression of *BG1* and *ABI4*, respectively.

Like the ABI3 and ABI5 transcription factors, ABI4 also plays a critical role in ABA responses, but compared with ABI3 and ABI5, how the activity of ABI4 is modulated is largely unknown. In this study, we found that HD-ZIPIII transcription factors, which are the direct targets of miR165/166, could directly bind to the *ABI4* gene promoter and regulate its expression. Thus, miR165/166 is relevant to *ABI4* in ABA responses. It has been observed that the expression of miR165/166 was altered under different abiotic stress conditions, such as cold, heat, salt and oxidative stress [66–70]. Regulation of miR165/166 expression may help plants to cope with environmental stresses. Given that miR165/166 is an important regulator in plant growth and development, the miR165/166 meditated regulatory module might help coordinate developmental programs with environmental cues to optimize plant growth and developmental processes under stress. miR165/166 is evolutionarily conserved in a wide range of plant species and its function in plant development is also very conserved. Future studies will determine whether the role of miR165/166 mediated regulatory module in ABA response and homeostasis is conserved in other plant species.

## Materials and Methods

### Plant materials and growth conditions

All *Arabidopsis* plants used in this study are in the Columbia-0 (Col-0) ecotype. Plants were grown in soil at 23°C under a 16h light/8h dark cycle. STTM165/166, *PHB*:*PHB G202G-YFP* lines, *bg1-2* and *abi4-1* have been described previously [10, 65, 71, 72].

### Plasmid construction

To construct *GST-PHB*, the DNA of *PHB* was amplified using the genomic DNA of *PHB*:*PHB G202G-YFP* lines as template. The PCR products were purified and digested with EcoRI and inserted into the corresponding sites of the pGEX4T-1 vector.

### Germination assay

To measure the rate of germination, seeds were harvested and stored under identical conditions. Seeds were surfaced sterilized and stored at 4°C for 3 days. Seeds were plated on MS plates containing 1% sucrose and 0.3% phytogel, and germinated at 22°C in a 16-h/8-h light/dark condition.

### Freezing tolerance assay

Electrolyte leakage assay was performed as previously described [73] to determine the freezing tolerance of plants in this study. In brief, 3-week-old plants grown in soil were subjected to cold acclimation at 4°C for 7 days before freezing treatment. At each temperature point, three replicates were performed. A fully developed rosette leaf was placed in a small tube containing 100 ul deionized water, and a small ice chip was then added to each tube. Incubate the tube in a freezing bath (model 1187, VWRScientific) with temperature at 0°C. The temperature was reduced by 1°C every 30 min until -11°C was reached. At each temperature point, the tubes were removed from the freezing bath and placed on ice. Transfer the leaves and solutions to large tubes with 25 ml deionized water. Shake the tubes overnight and measure the conductivity of solutions. Then autoclave the tubes at 121°C around 20 min, and shake the tubes for another 3 hours before measuring the conductivity. Finally, calculate the ratio of conductivity before and after autoclaving.

For freezing survival assay, 12-day-old seedlings grown on MS plates containing 1% sucrose and 0.8% agar were subjected to cold acclimation at 4°C for 7 days. The freezing treatment was conducted in a freezing chamber with the following program: the temperature was set at 4°C and reduced to 0°C within 30 min and then the temperature was reduced 1°C every 1hr until -7°C was reached. Transfer the plates at 4°C for 12hr in the dark and recover the seedlings at 23°C for 5 days.

### ABA treatment

For ABA treatment, seedlings were grown in ½ MS liquid medium for one week were treated with 50 μm ABA for the indicated times as described previously [74].

### Real-time RT-PCR analysis

For the examination of mRNA expression level, total RNA was extracted using the RNeasy mini kit (Qiagen) according to the manufacturer’s instructions, and reversely transcribed using the High-Capacity cDNA Archive Kit (Applied Biosystems). Quantitative real-time PCR was performed using the SYBR Green PCR master mix kit according to the manufacturer’s instructions. Actin mRNA was used as an internal control. Relative gene expression level was calculated from 2-ΔΔCt values. Primers used for qPCR are listed in S1 Table.

### Mature miRNA quantification

Mature miRNA quantification was performed according to TaqMan Small RNA Assays protocol (Applied Biosystems). Arabidopsis SnoR101 was used as an internal control. TaqMan Gene Expression Master Mix (Applied Biosystems) was used to perform qRT-PCR.

### Chromatin Immunoprecipitation (ChIP) assays

ChIP assays were conducted as previously described [75]. Briefly, 2.0 g materials and the anti-GFP (Abcam) antibody were used for ChIP assay. The precipitated DNA was dissolved in 100 ul of TE buffer, and 2 ul was used for ChIP real-time PCR. Three independent biological replicates were performed, and a representative result is presented. Primer pairs used for ChIP enrichment test are described in S1 Table.

### Electrophoretic Mobility Shift Assay (EMSA)

GST-PHB recombinant fusion protein was expressed in the *E*. *coli* BL21 strain and purified using Glutathione sepharose 4B beads (GE Healthcare). The oligonucleotides were labeled with α-^32^P-dATP using T4 Polynucleotide Kinase (NEB), the ^32^P-labeled probes were incubated in 20 ul reaction mixtures containing 20 mM Tris-HCI (pH7.5), 300 mM NaCI, 5 mM MgCI_2_, 0.1% NP-40, 0.5 mM DTT for 20 to 60 min at room temprature, and separated on 6% polyacrylamide gels in Tris-glycine buffer (50 mM Tris, 380 mM glycine, 2 mM EDTA, pH 8.0). The oligonucleotides used for EMSA are listed in S1 Table.

### Measurement of endogenous ABA levels

ABA content was measured with a Phytodetek ABA test kit (Agdia, Inc., Elkhart, IN) following the manufacturer’s instructions.

## Supporting Information

S1 Fig The phenotype of wild type and STTM165/166 plants grown on MS medium supplemented with 0.1 μM ABA. Seedlings were photographed 7 days after stratification.
(TIF)

S2 Fig Quantitative RT-PCR analysis of the expression of both mature miR165/166 and its targets of 2-day-old seedlings grown on the MS medium containing 1.0 μM ABA. Three independent experiments were performed, and values are means ± standard deviation. Values are means ± standard deviation.
(TIF)

S3 Fig The phenotype of wild type and *phb-13phv-11* plants grown on MS medium supplemented with 1.0 μM and 2.0 μM ABA. Seedlings were photographed 9 days after stratification.
(TIF)

S4 Fig Comparison of the expression of ABA-responsive genes in wild type and STTM165/166 seedlings treated with 50 μM ABA with a time course. Transcript abundance of *RD29B* and *RAB18* was analyzed using qRT-PCR. Three independent experiments were performed, each with three replicates. Values are means ± standard deviation.
(TIF)

S5 Fig Comparison of the expression of core components of the ABA signaling pathway in wild type and STTM165/166 seedlings. Transcript abundance of genes involved in ABA signaling pathway was analyzed using qRT-PCR. Three independent experiments were performed, each with three replicates. Values are means ± standard deviation.
(TIF)

S6 Fig Comparison of the expression of core components of the ABA signaling pathway, *PYL*s, in wild type and STTM165/166 seedlings treated with 50 μM ABA for different time periods. Transcript abundance of *PYL*s was analyzed using qRT-PCR. Three independent experiments were performed, each with three replicates. Values are means ± standard deviation.
(TIF)

S7 Fig Comparison of the expression of other core components of the ABA signaling pathway, *ABI1*, *ABI2* and *HAB1*, in wild type and STTM165/166 seedlings treated with 50 μM ABA for different time periods. Transcript abundance of *ABI1*, *ABI2* and *HAB1* was analyzed using qRT-PCR. Three independent experiments were performed, each with three replicates. Values are means ± standard deviation.
(TIF)

S8 Fig Comparison of the expression of other core components of ABA signaling pathway, *SnRK2*.*2*, *SnRK2*.*3* and *SnRK2*.*6*, in wild type and STTM165/166 seedlings treated with 50 μM ABA for different time periods. Transcript abundance of *SnRK2*.*2*, *SnRK2*.*3* and *SnRK2*.*6* was analyzed using qRT-PCR. Three independent experiments were performed, each with three replicates. Values are means ± standard deviation.
(TIF)

S9 Fig Comparison of the expression of genes involved in de novo ABA biosynthesis in wild type and STTM165/166 seedlings. Transcript abundance of genes involved in de novo ABA biosynthesis was analyzed using qRT-PCR. Three independent experiments were performed, each with three replicates. Values are means ± standard deviation.
(TIF)

S10 Fig Comparison of the phenotypes of wild type, STTM165/166 and STTM165/166 in *bg1-2* background under drought conditions. (A) Drought resistance test. 3-week-old plants (upper panel) were grown under the same conditions but without irrigation for 12 days (middle panel), and then re-watered for 3 days (lower panel). (B) Quantification of survival rates. Thirty plants of wild type and STTM165/166 were used in each experiment, and the survival rate was calculated from the results of four independent experiments. (C) Water loss assay. Aerial parts of 3-week-old plants were detached and weighed at the indicated time points.Water content at any time point was calculated as percentage of the fresh weight at time zero. Data were derived from four independent experiments (±SD).
(TIF)

S1 Table Primers and oligonucleotides used in this study. (XLSX)
