# Supplementary figures and images for: The miR165/166 Mediated Regulatory Module Plays Critical Roles in ABA Homeostasis and Response in Arabidopsis thaliana
Source: PLoS Genet. 2016 Nov 3;12(11):e1006416. doi: 10.1371/journal.pgen.1006416 (PMC5094776; doi:10.1371/journal.pgen.1006416)

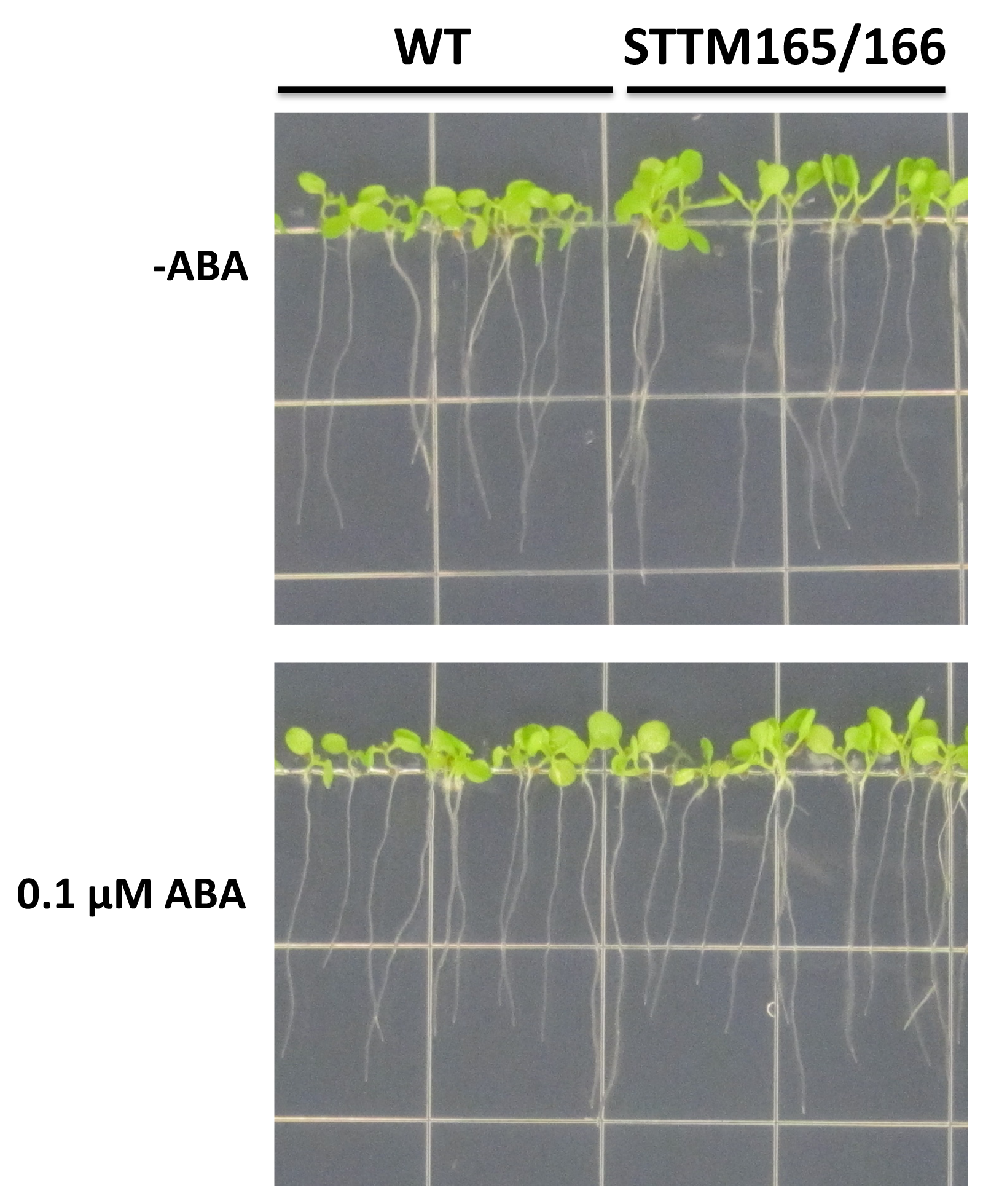

Supplement: S1 Fig — Seedlings were photographed 7 days after stratification. (TIF) [file pgen.1006416.s001.tif]

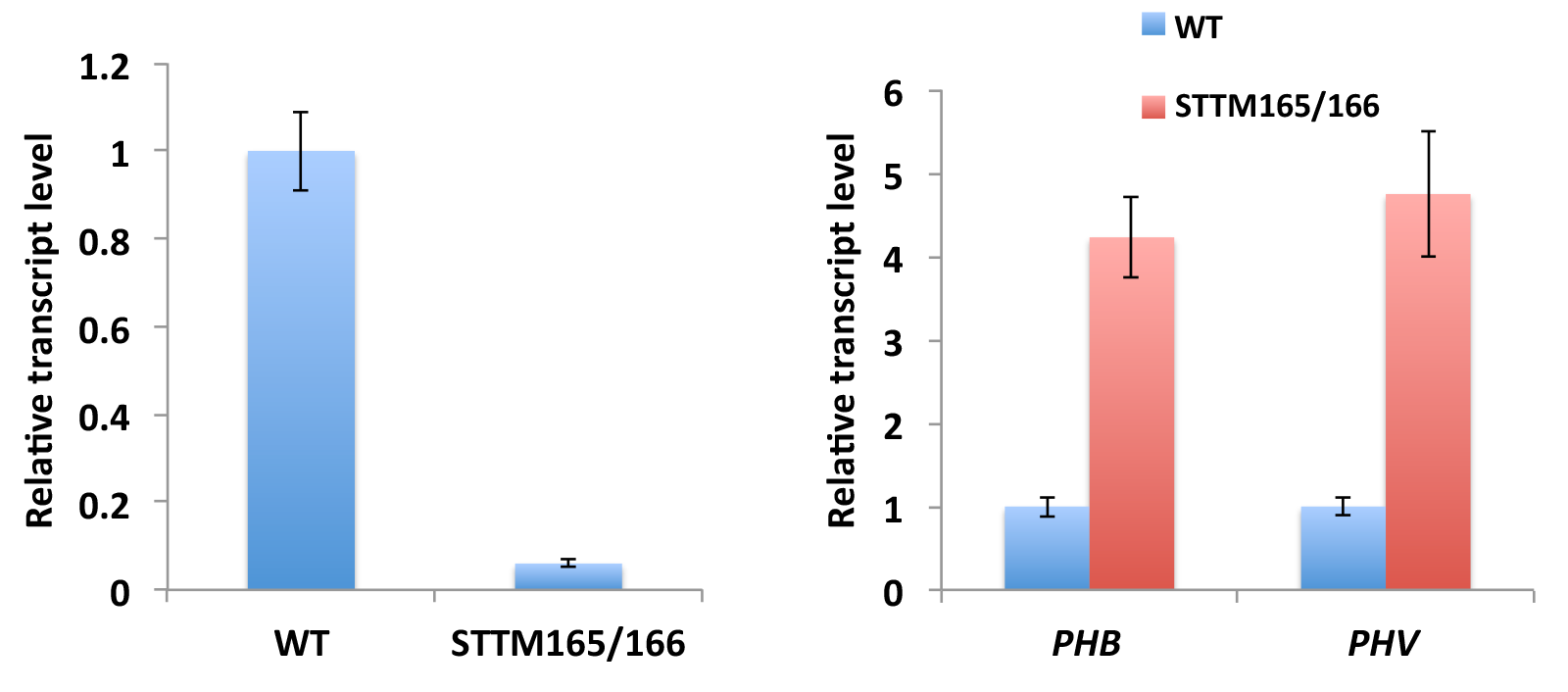

Supplement: S2 Fig — Three independent experiments were performed, and values are means ± standard deviation. Values are means ± standard deviation. (TIF) [file pgen.1006416.s002.tif]

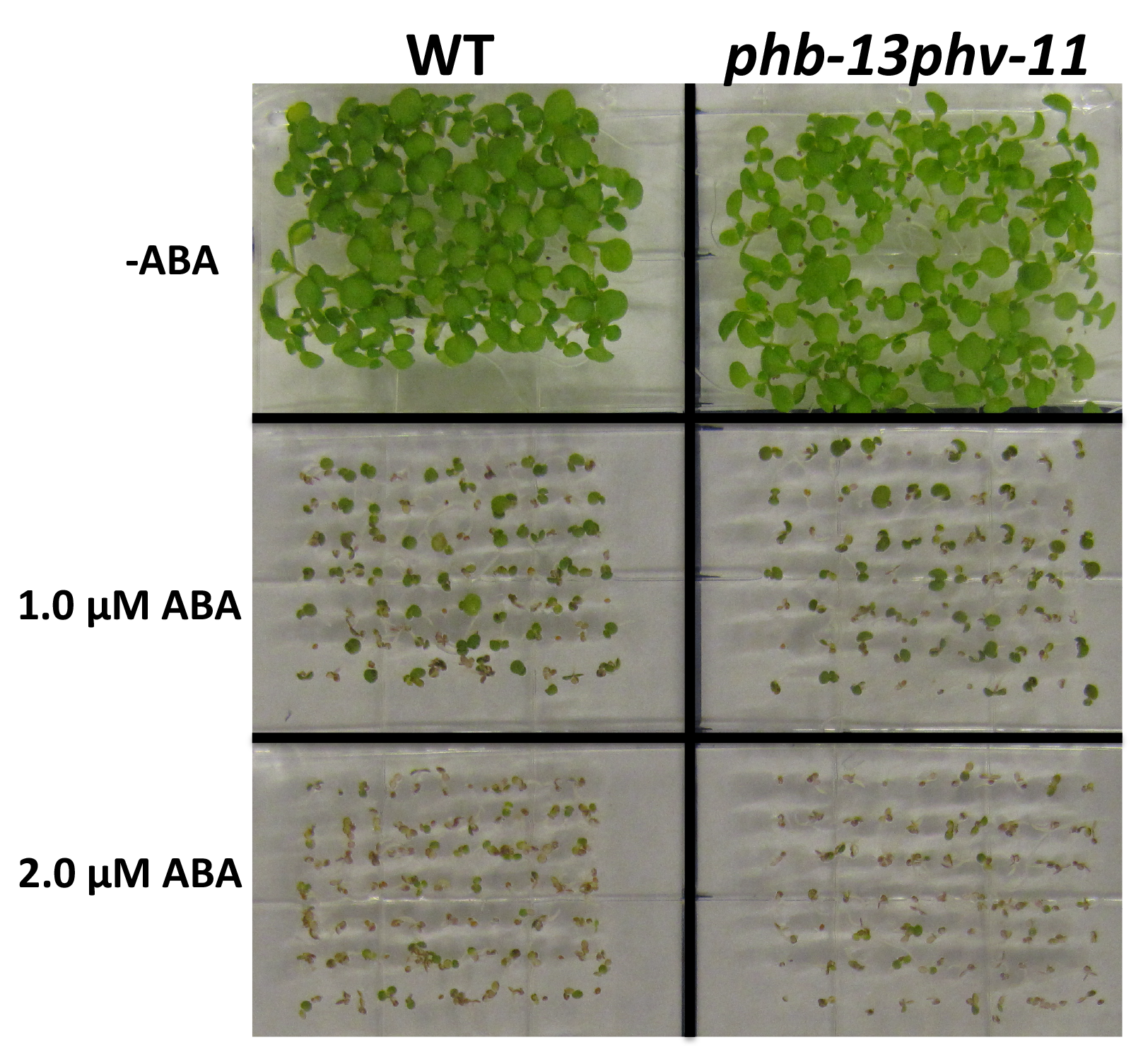

Supplement: S3 Fig — Seedlings were photographed 9 days after stratification. (TIF) [file pgen.1006416.s003.tif]

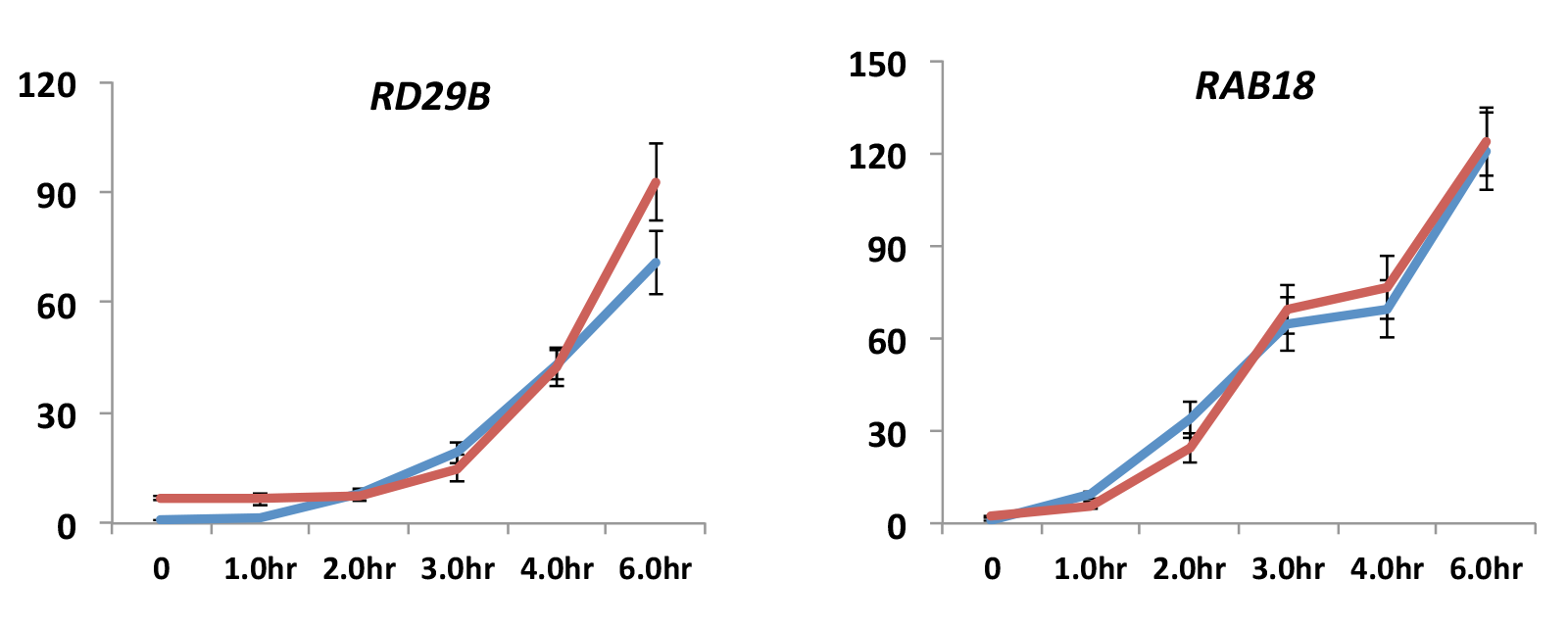

Supplement: S4 Fig — Transcript abundance of RD29B and RAB18 was analyzed using qRT-PCR. Three independent experiments were performed, each with three replicates. Values are means ± standard deviation. (TIF) [file pgen.1006416.s004.tif]

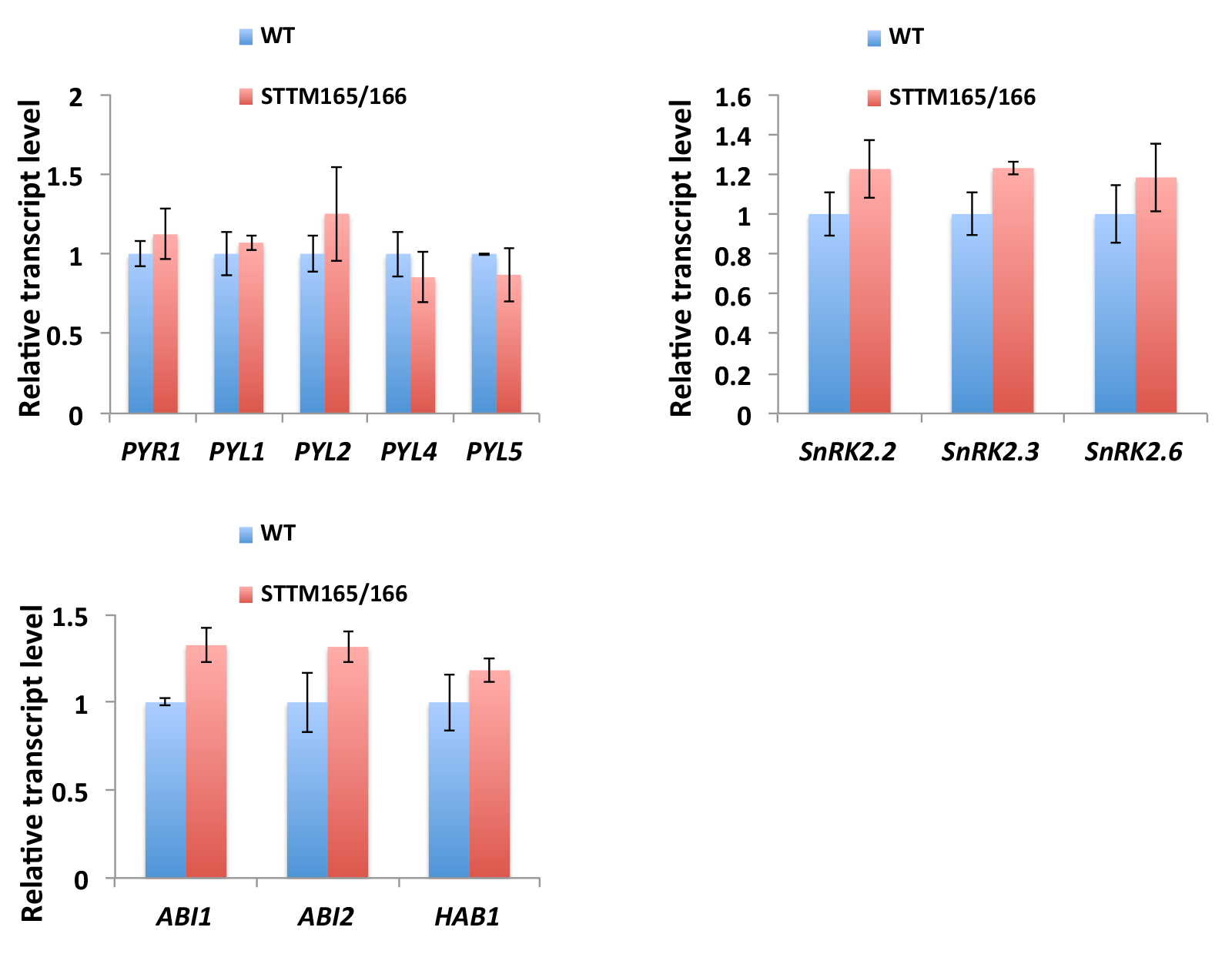

Supplement: S5 Fig — Transcript abundance of genes involved in ABA signaling pathway was analyzed using qRT-PCR. Three independent experiments were performed, each with three replicates. Values are means ± standard deviation. (TIF) [file pgen.1006416.s005.tif]

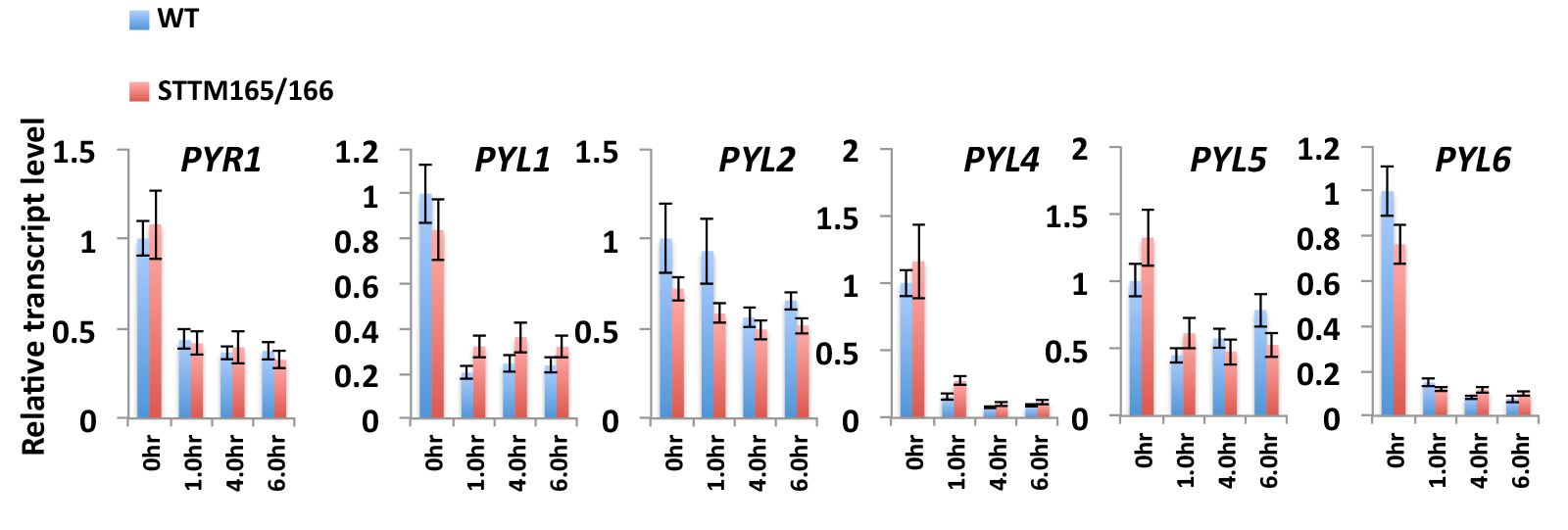

Supplement: S6 Fig — Transcript abundance of PYLs was analyzed using qRT-PCR. Three independent experiments were performed, each with three replicates. Values are means ± standard deviation. (TIF) [file pgen.1006416.s006.tif]

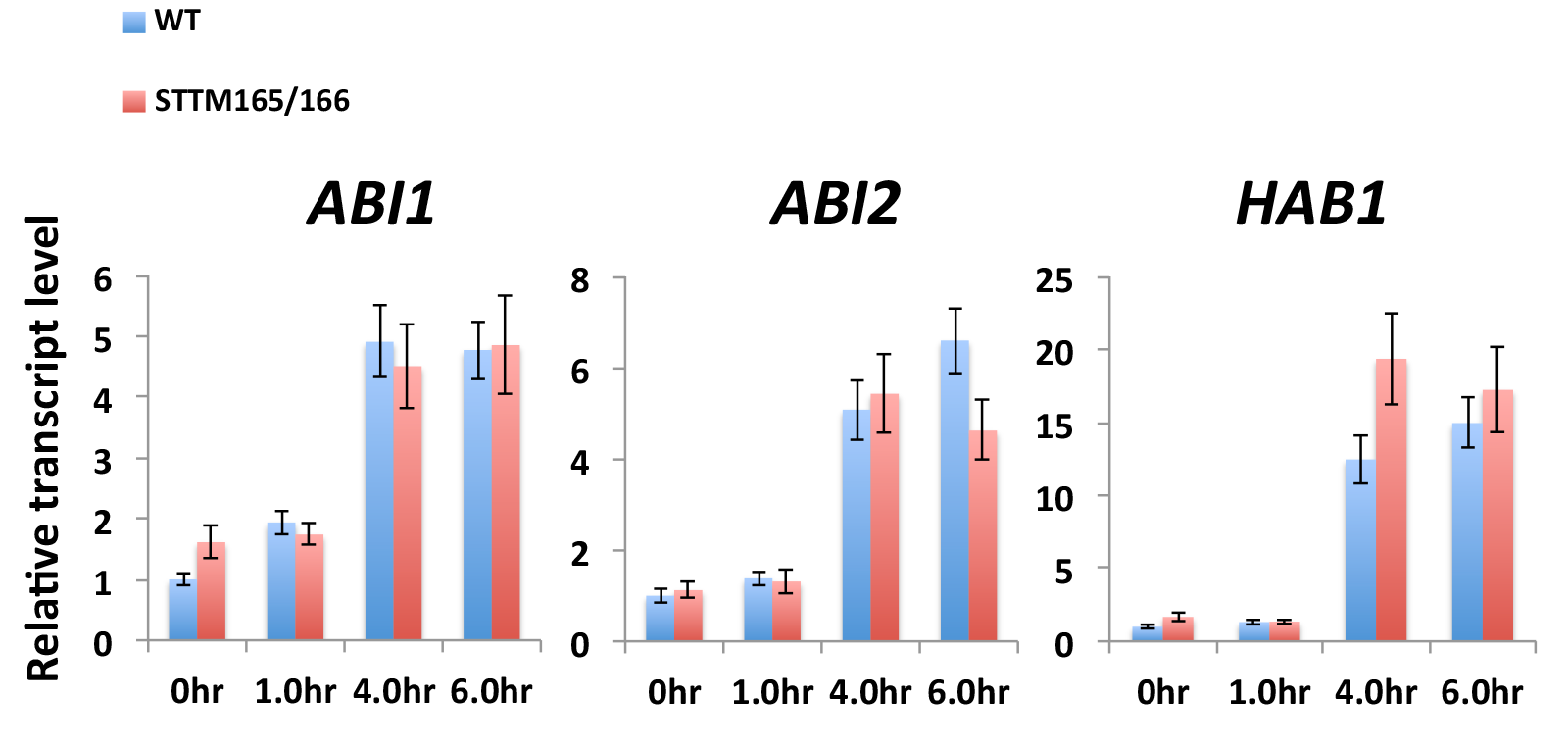

Supplement: S7 Fig — Transcript abundance of ABI1, ABI2 and HAB1 was analyzed using qRT-PCR. Three independent experiments were performed, each with three replicates. Values are means ± standard deviation. (TIF) [file pgen.1006416.s007.tif]

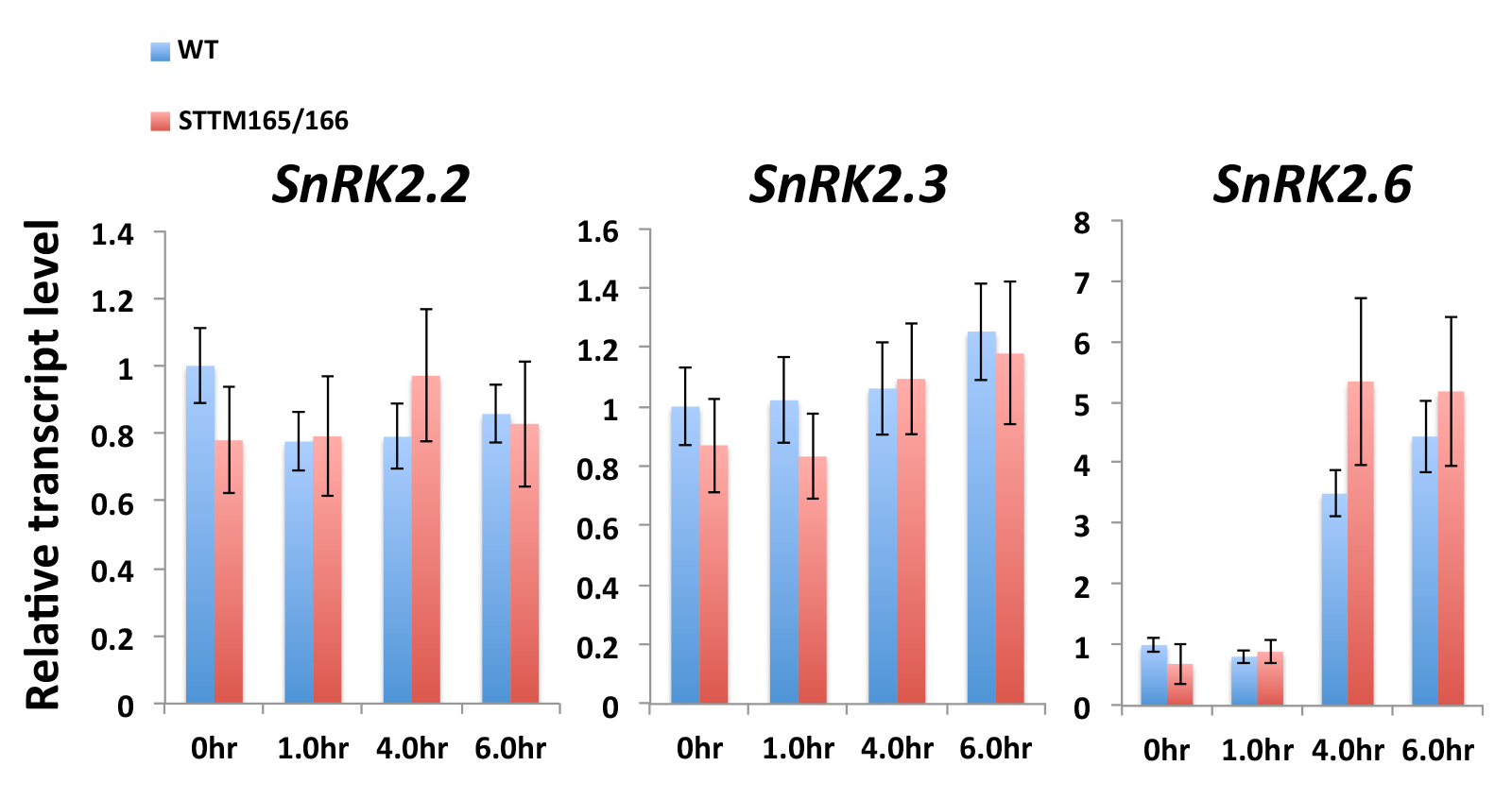

Supplement: S8 Fig — Transcript abundance of SnRK2.2, SnRK2.3 and SnRK2.6 was analyzed using qRT-PCR. Three independent experiments were performed, each with three replicates. Values are means ± standard deviation. (TIF) [file pgen.1006416.s008.tif]

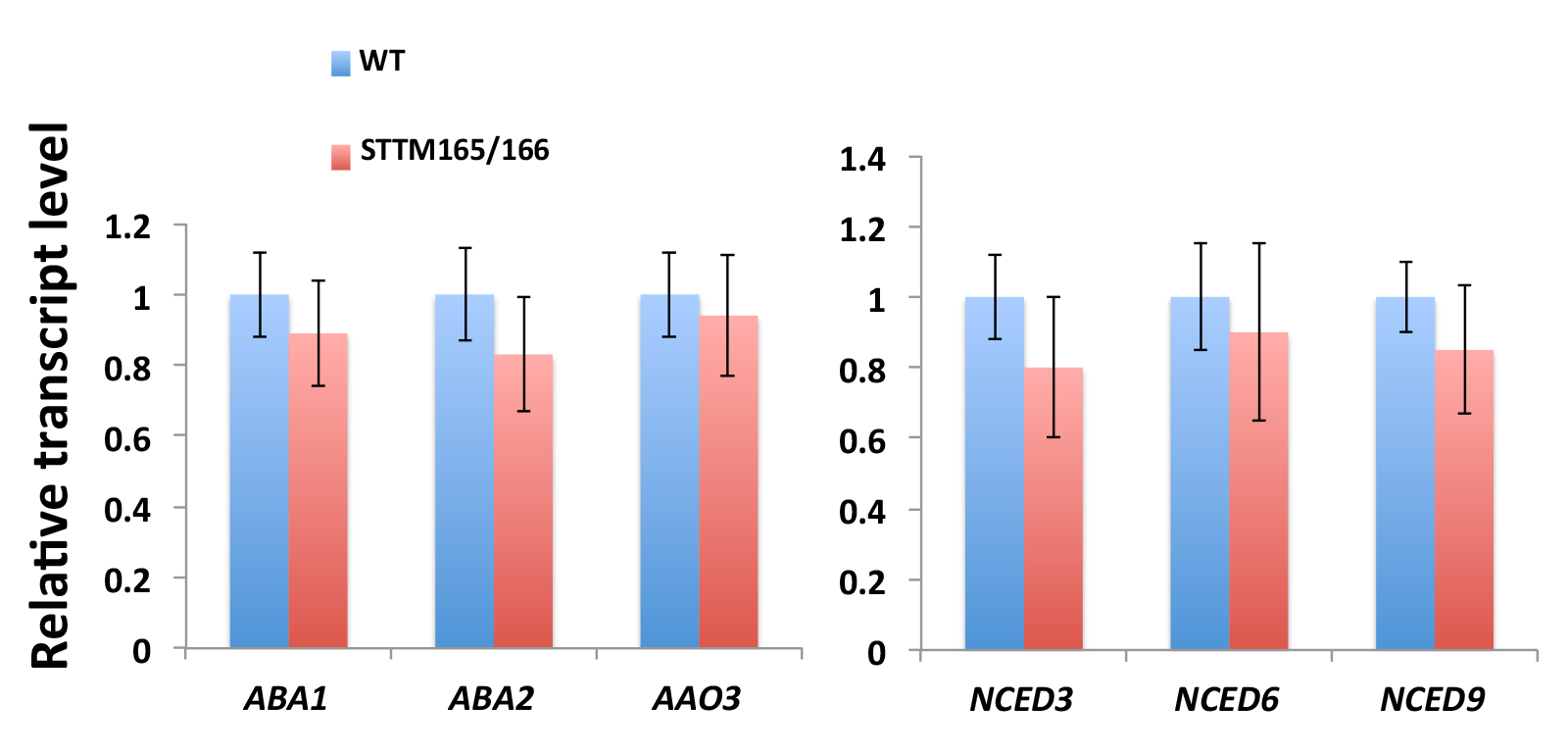

Supplement: S9 Fig — Transcript abundance of genes involved in de novo ABA biosynthesis was analyzed using qRT-PCR. Three independent experiments were performed, each with three replicates. Values are means ± standard deviation. (TIF) [file pgen.1006416.s009.tif]

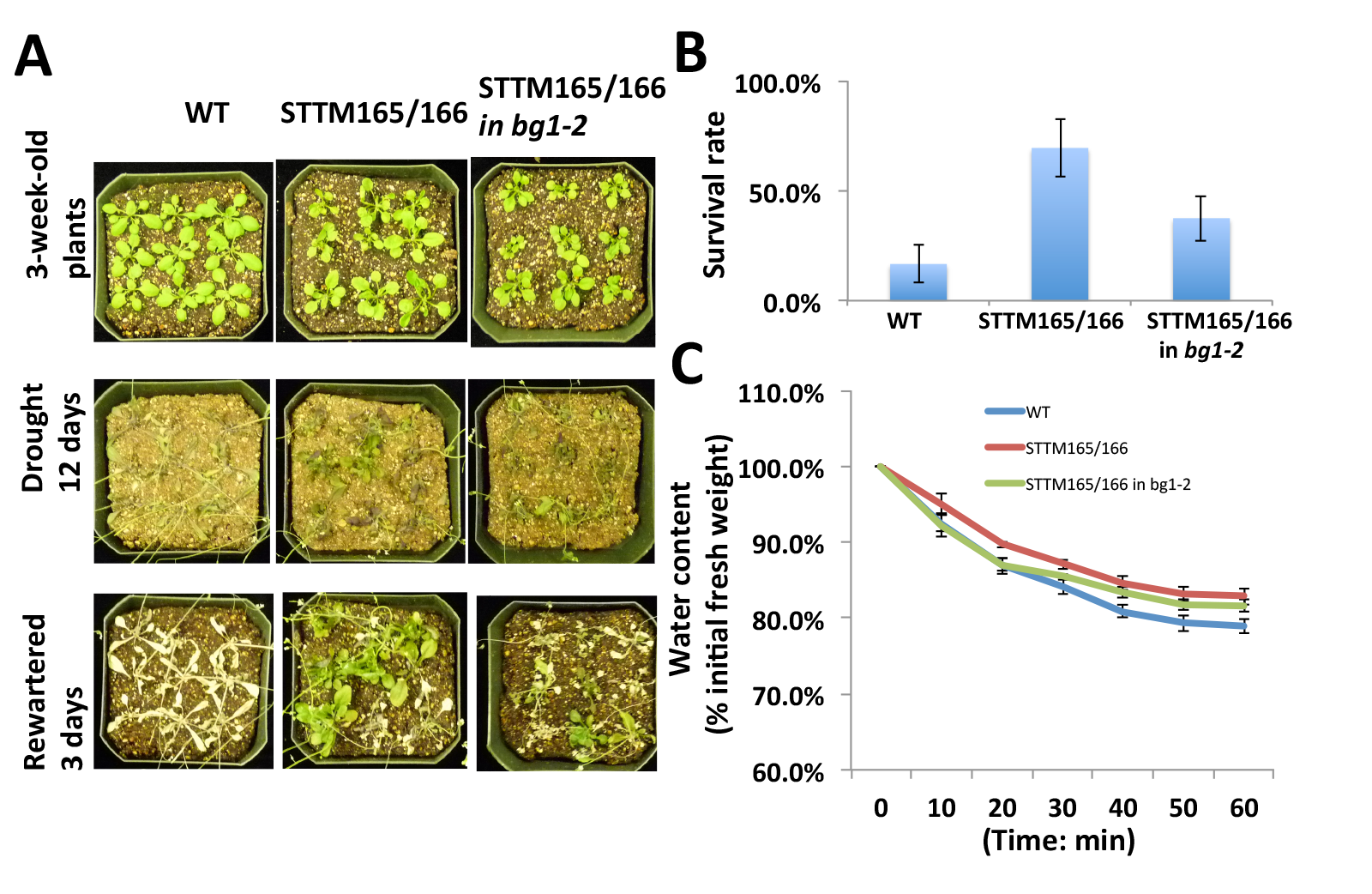

Supplement: S10 Fig — (A) Drought resistance test. 3-week-old plants (upper panel) were grown under the same conditions but without irrigation for 12 days (middle panel), and then re-watered for 3 days (lower panel). (B) Quantification of survival rates. Thirty plants of wild type and STTM165/166 were used in each experiment, and the survival rate was calculated from the results of four independent experiments. (C) Water loss assay. Aerial parts of 3-week-old plants were detached and weighed at the indicated time points.Water content at any time point was calculated as percentage of the fresh weight at time zero. Data were derived from four independent experiments (±SD). (TIF) [file pgen.1006416.s010.tif]
